# A New Synthetic Peptide Having Two Target of Antibacterial Action in *E. coli* ML35

**DOI:** 10.3389/fmicb.2016.02006

**Published:** 2016-12-20

**Authors:** Adriana Barreto-Santamaría, Hernando Curtidor, Gabriela Arévalo-Pinzón, Chonny Herrera, Diana Suárez, Walter H. Pérez, Manuel E. Patarroyo

**Affiliations:** ^1^Receptor-Ligand Department, Fundación Instituto de Inmunología de ColombiaBogotá, Colombia; ^2^Faculty of Sciences and Education, Universidad Distrital Francisco José de CaldasBogotá, Colombia; ^3^School of Medicine and Health sciences, Universidad del RosarioBogotá, Colombia; ^4^Escuela Colombiana de Carreras IndustrialesBogotá, Colombia; ^5^Faculty of Medicine, Universidad Nacional de ColombiaBogotá, Colombia

**Keywords:** antimicrobial peptide (AMP), synthetic peptide, minimum inhibitory concentration (MIC), liposome, membrane phospholipid, membrane permeabilization

## Abstract

The increased resistance of microorganisms to the different antimicrobials available to today has highlighted the need to find new therapeutic agents, including natural and/or synthetic antimicrobial peptides (AMPs). This study has evaluated the antimicrobial activity of synthetic peptide 35409 (RYRRKKKMKKALQYIKLLKE) against *Staphylococcus aureus* ATCC 29213, *Pseudomonas aeruginosa* ATCC 15442 and *Escherichia coli* ML 35 (ATCC 43827). The results have shown that peptide 35409 inhibited the growth of these three bacterial strains, having 16-fold greater activity against *E. coli* and *P. aeruginosa*, but requiring less concentration regarding *E. coli* (22 μM). When analyzing this activity against *E. coli* compared to time taken, it was found that this peptide inhibited bacterial growth during the first 60 min and reduced CFU/mL 1 log after 120 min had elapsed. This AMP permeabilized the *E. coli* membrane by interaction with membrane phospholipids, mainly phosphatidylethanolamine, inhibited cell division and induced filamentation, suggesting two different targets of action within a bacterial cell. Cytotoxicity studies revealed that peptide 35409 had low hemolytic activity and was not cytotoxic for two human cell lines. We would thus propose, in the light of these findings, that the peptide 35409 sequence should provide a promising template for designing broad-spectrum AMPs.

## Introduction

Antibiotics are molecules combating part of the infections produced by bacteria. However, the appearance of resistant strains, such as vancomycin-resistant *Staphylococcus aureus*, methicillin-resistant *Staphylococcus epidermidis*, ampicillin-resistant and carbapenemase-resistant *Escherichia coli*, has become a global public health problem and driven the search for new therapeutic compounds having antimicrobial activity which can counteract this phenomenon (Rodriguez-Noriega et al., 2010; Elhani et al., 2012; Kaase et al., 2016). This has led to discovering and isolating natural antimicrobial peptides (AMPs) and developing synthetic peptides having antimicrobial activity and improved selectivity (Broekaert et al., 1995; Frecer et al., 2004; Chen et al., 2005; Jenssen et al., 2006; Bea Rde et al., 2015).

Antimicrobial peptides have a broad spectrum of activity against fungi, parasites, viruses, and bacteria (Gram-positive and Gram-negative) (Koczulla and Bals, 2003; Bulet et al., 2004; Reddy et al., 2004). Most of them share common characteristics, such as length (12-100 residues), positive net charge and amphipathic structures; however; they have little sequence homology and a broad range of secondary structures (Lewies et al., 2015). AMPs’ most important mechanism of action lies in altering membrane organization and depolarization through electrostatic and hydrophobic interactions with negatively charged lipids on cell membrane (Yeaman and Yount, 2003; Reddy et al., 2004; Teixeira et al., 2012). It has been described that AMPs can exercise their activity through peptide-membrane interactions, cell entry and binding to intracellular molecules, and inhibiting the synthesis of enzymes from cell wall, DNA, RNA, or proteins (Peters et al., 2010; Lewies et al., 2015). Additionally, AMP activity and mechanism of action have been related to their amino acid sequence, concentration, net charge, secondary structure, hydrophobicity, as well as the bacterial membrane composition (Dathe et al., 1997; Epand et al., 2005; Spindler et al., 2011).

Antimicrobial peptides have been classified into four main groups based on their structure and composition: alpha-helix peptides such as cecropin-A (Jenssen et al., 2006), beta-sheet peptides (i.e., human β-defensin-1) (Broekaert et al., 1995), mixed structure (i.e., plectasin) (Frecer et al., 2004) and specific amino acid-rich peptides, such as indolicidin having a large amount of tryptophan (Falla and Hancock, 1997). Regarding such classification, it has been reported that alpha-helix amphipathic peptides are usually more active than those having less-defined secondary structures (Brogden, 2005).

Several studies have focused on natural AMPs isolated from different animal species, for example cecropins isolated from insects mainly having activity against Gram-negative bacteria (Steiner et al., 1981), magainin isolated from frog skin (*Xenopus laevis*) having activity against Gram-positive and Gram-negative bacteria (Zasloff, 1987) and dermaseptin isolated from tree frog skin having action on a wide spectrum of microorganisms, such as protozoa, bacteria, yeast, and filamentous fungi (Mor et al., 1994; Ghosh et al., 1997). Unfortunately, some of the greatest problems involved in using native AMPs as therapeutic components is their high toxicity and their ability to lyse eukaryotic cells (Koczulla and Bals, 2003). Nevertheless, some studies have revealed that selective modification in such peptide sequences (e.g., reducing length, modifying structure, replacing amino acids, and fusion with other sequences) has led to notably reducing toxic activity against eukaryotic cells whilst maintaining or increasing their antimicrobial activity (Sitaram et al., 1992; Navon-Venezia et al., 2002; Zelezetsky and Tossi, 2006; Almaaytah et al., 2012). Designing synthetic AMPs (imitating/mimicking physical–chemical properties from native AMPs) (Joshi et al., 2010) or native AMPs analogous peptides has opened up a research field into new synthetic molecules only having activity against prokaryotic cells (Falla and Hancock, 1997; Fox et al., 2012).

Peptide 35409 (RYRRKKKMKK**A**LQYIKLLKE) is a new peptide analog from peptide 20628 (^321^RYRRKKKMKKKLQYIKLLKE^340^), in turn, derived from the *Plasmodium falciparum Pf*Rif protein (Weber, 1988). *Pf*Rif forms part of the family of proteins called Rifins which are characterized by their low molecular weight (30-45 KD) and expression during different parasite stages (sporozoite, merozoites, and gametes) (Florens et al., 2002). Rifins are clonally variant antigens expressed on infected red blood cells (iRBCs), associated with the pathogenesis of malaria by cytoadhesion (rosetting) and evasion of the immune response (Kyes et al., 1999; Petter et al., 2007; Wang and Hviid, 2015).

In the search for high activity binding peptides as anti-malarial vaccine candidates (Patarroyo and Patarroyo, 2008; Rodriguez et al., 2008) it was found that peptide 20628 caused the lysis of human RBC (10.4% at 200 μM) (unpublished data). Peptide 20628 did not inhibit Gram-negative (*E. coli* ATCC 25922) or Gram-positive (*S. aureus* ATCC 29213) bacterial growth whilst its analog 35409 (K331A) had reduced hemolytic activity and inhibited *E. coli* and *S. aureus* bacterial growth (Maya, 2009).

Comparing peptide 35409 sequence to AMP database sequences (collecting, predicting, and classifying AMPs) (Lata et al., 2010) showed that peptide 35409 could have had antibacterial activity, this being similar to previously described AMPs (e.g., 39.28% similarity with natural latarcin 1 AMP isolated from the poisonous spider *Lachesana tarabaevi*) (Kozlov et al., 2006; Rothan et al., 2014). Furthermore, peptide 35409 had arginine in position 1, and this has been reported as being one of the preferential residues towards the amino-terminal region of some AMPs (Lata et al., 2007).

The present work aimed at characterizing peptide 35409 antimicrobial activity concerning different types of bacteria and their mechanism of action against *E. coli* ML35. The results showed that peptide 35409 had antibacterial activity against *Escherichia coli* ML35 and *Pseudomonas aeruginosa* ATCC 15442 at low concentrations and that this peptide did not affect eukaryotic cell viability and maintained low hemolysis percentages. Our results suggested that peptide 35409 permeabilized *E. coli* ML35 membrane through its interaction with phosphatidylethanolamine (PE) (a phospholipid component present in high concentrations on bacterial membrane), thereby enabling peptide molecule entry to a cell where it interacts with the DNA, inhibiting its synthesis and consequently bacterial cell division.

## Materials and Methods

### Peptide Synthesis and Purification

*Pf-Rif* 20628 (^321^RYRRKKKMKKKLQYIKLLKE^340^): 35409 (RYRRKKKMKK**A**LQYIKLLKE) (K331A), and 35415 (RYRRKKKMKKKLQYIK**A**LKE) (K337A) peptide analogs were synthesized using the solid phase t-Boc strategy on MBHA resin (0.5 meq/g) (Merrifield, 1969). Lyophilized peptides were analyzed by reverse-phase high-performance liquid chromatography (RP-HPLC) on a Merck-Hitachi chromatograph on a C-18 column in a 0-70% acetonitrile linear gradient for 45 min at 250 μL/min flow-rate, greater than 90% purity being determined. Synthesized peptides’ molecular mass was determined by MALDI-TOF mass spectrometry on Microflex equipment (Bruker) using α-Cyano-4-hydroxycinnamic acid (Sigma) as matrix. The same methodology was used for synthesizing cecropin (KWKVFKKIEKMGRNIRNGIVKAGPAIAVLGEAKAL) (Steiner et al., 1981) and scrambled (same amino acid composition but different sequence) peptide 38659 (YKLQLKRKREKKIYMRKKLA) designed with Shuffle Protein software from peptide 35409 sequence. Cecropin and peptide 38659 were used as positive and negative controls, respectively.

### Circular Dichroism (CD)

The peptides’ secondary structure was examined by CD. The peptides (5 μM) were analyzed using a 1-cm light pass length quartz cell thermostated at 20°C using 30% (v/v) 2,2,2- trifluoroethanol (TFE) as co-solvent as it has been shown to stabilize secondary structures (Buck, 1998; Povey et al., 2007). Spectra were obtained on a nitrogen-flushed Jasco J-810 spectrometer at room temperature by averaging three sweeps taken from 260 to 190 nm at a 20 nm/min scan rate and 1 nm bandwidth. Data was collected using Spectra Manager Software and analyzed using SELCON3, CONTINLL, and CDSSTR software, as reported previously (Sreerama et al., 1999).

### Measuring Antibacterial Activity

Minimal inhibitory concentration (MIC) was determined using standard micro-titer dilution, standard techniques for determining peptide, and antibiotic antimicrobial activity approved by the Clinical and Laboratory Standards Institute (CLSI) (Wiegand et al., 2008). Briefly, cells were grown overnight in Luria-Bertani (LB) agar at 37°C. Morphologically similar colonies (3-4) were used for inoculating 5 mL LB liquid medium. Following 4-5 h growth (∼1 × 10^8^ colony-forming unit CFU), the bacteria were harvested by spinning at 685 × *g* for 20 min, washed twice with PBS, pH 7.2 at 4°C and diluted in fresh PBS until an initial 5 × 10^6^ CFU/mL working inoculum was obtained (Wiegand et al., 2008). Optical density (OD) was read at 620 nm and precise amounts of bacteria were measured as OD_620_ = 0.2 = 5 × 10^7^ CFU/mL (Hiemstra et al., 1993).

Serial peptide dilutions, bacterial inoculum (15 μL) and media were added to the micro-titer plates (150 μL final volume) and incubated for 18 h at 37°C. MIC was determined as being the lowest peptide concentration that inhibited growth by measuring OD_620_. Cecropin-treated cells and cells without peptides were used as positive and negative controls, respectively. Sterile LB medium was used as sterility control. Assays were carried out in duplicate. *S. aureus* ATCC 29213, *P. aeruginosa* ATCC 15442, and *E. coli* ML 35 (ATCC 43827) were the bacterial strains used.

### Bactericidal Kinetics

Peptide 35409 bactericidal kinetics was evaluated by incubating peptide (MIC concentration) with *E. coli* (5 × 10^5^ CFU/mL). Peptide/bacteria mixtures (100 μL) were taken at 0, 30, 60, 90, and 120 min and serially diluted. These dilutions were plated on LB agar and incubated for 18 h to determine cell viability and the number of CFU/mL. Data was obtained from three independent experiments performed in duplicate. Bacteria in the absence of peptide were taken as control (Lehrer et al., 1983).

### Peptide 35409 Action on *E. coli* ML35 Membrane

Peptide 35409 activity on *E. coli* ML35 bacterial membrane was studied by three different techniques. Scanning electron microscopy was used for describing morphological changes regarding *E. coli* or *E coli*-derived spheroplasts after incubation with peptide 35409. Flow cytometry was used for evaluating peptide permeabilization capability related to cytoplasmatic membrane whilst ortho-nitrophenyl-β-galactoside (ONPG) hydrolysis assay was used for studying permeabilization concerning time taken.

#### Scanning Electron Microscopy (SEM)

*Escherichia coli* ML35 strain spheroplasts were obtained by 1% lysozyme treatment, following previously described methodology (Zerrouk et al., 2008). SEM involved taking spheroplasts or 5 × 10^5^ UFC/mL *E. coli* ML35 grown as mentioned in Section “Measuring Antibacterial Activity” and incubated with peptide 35409 for 1 h at 37°C in LB liquid medium (22 μM final concentration). They were then washed with 1X PBS and bacteria or spheroplasts were fixed with 2.5% glutaraldehyde. The samples were dehydrated using ethanol at a range of concentrations from 70 to 100% and critical points were dried in EK3150 drier. The samples were then gold coated, using the Quorum Q150R ES coating system, and analyzed by Phenom scanning electron microscope, 100× at 10 KV. Bacteria or spheroplasts without peptide were used as negative control [protocol adapted from (Hartmann et al., 2010)].

#### Flow Cytometry

*Escherichia coli* bacteria (5 × 10^5^ CFU/mL) were incubated with 88 μM (4 × MIC) peptide 35409 (500 μL final volume) for 3 h at 37°C. The peptide–bacteria mixture was then incubated with 3 μL 25% propidium iodide (PI) for 15 min in the dark (Chau et al., 2011). Fluorescence was read by FACSCanto II (Beckton Dickinson) flow cytometer (4-2-2 configuration) using an FL2-H filter and FACSDiva software (Beckton Dickinson) was used for analyzing the data. Bacteria treated with cecropin (12 μM) were used as membrane permeabilization control. Dead bacteria obtained by heat treatment (5 min at 100°C and 3 h at 70°C) and bacteria without any treatment were used for establishing cut-off points between bacteria having permeabilized membranes and living ones.

#### ONPG Hydrolysis

Inner membrane permeabilization was investigated by ONPG hydrolysis assay. *E. coli* ML-35 strain cells were grown to mid-log in LB liquid medium, washed twice with an equal volume of PBS and diluted in PBS to 5 × 10^7^ CFU/mL. Then, 15 μL of this solution (5 × 10^5^ CFU) was diluted with PBS (135 μL) supplemented with 1.5 mM ONPG and peptides 35409 (0, 11, 22, and 44 μM) or 35415 (22 μM). Maximum permeability was determined by evaluating cells pre-treated with cecropin (3 μM). Permeability rate was evaluated by ONPG hydrolysis, measuring absorbance at 405 nm in 30 min intervals for up to 3 h (Arcidiacono et al., 2009).

### Liposome Preparation

Large unilamellar vesicles (LUVs) were obtained for determining whether the membrane’s lipid composition affected peptide activity, according to a previously described methodology (Haginoya et al., 2005; Cheng et al., 2009). Briefly, L-α- PE (Sigma P7943) and L-α-phosphatidyl-DL-glycerol (PG) (Sigma P5531) lipids, singly or in mixture (8:2 PE: PG) (Epand et al., 2007), were dissolved in 5 mL dichloromethane. The solvent was evaporated in a Rotavapor at 450 mBar pressure at 60 rpm at 25°C until the appearance of a lipid film on the wall of the flask (left for 20–30 min more to ensure dryness).

The lipid film was dissolved with 1.5 mL buffer (150 mM NaCl, 0.1 g/L EDTA, 1 mM NaN3, 10 mM Tris-base) containing 10 mg/mL calcein and 0.25 M NaOH with strong shaking for 15 min. It was then left for 30 min at room temperature. The solution was passed 10 times through a 0.2 μm Nylon filter and left for 30 min for homogenization of vesicles and LUV formation. The liposomes were purified by size exclusion chromatography on a Sephacryl S300 HR column (0.5 cm × 20 cm). LUV size distribution was ascertained by SEM.

### Calcein Leakage Assay

Liposomes mimicking *E. coli* phospholipid composition (8:2 PE: PG) (Hancock and Lehrer, 1998; Epand et al., 2007), consisting solely of PE or PG, were used for the calcein release assay (Cheng et al., 2009; Fillion et al., 2015). The fluorescence of just liposomes or those incubated with peptide 35409 (22 μM) was monitored at different times over a 4 h period. Fluorescence was read on a Thermo-scientific Fluoroskan Ascent with 485 nm excitation and 538 nm emission filters. Liposomes were treated with 1 μL 20% Triton X-100 for determining maximum fluorescence intensity (taken as 100% calcein release) and percentage calcein release was calculated according to the following equation:

$$\%\;release=\frac{F-F_{0}}{F_{T}-F_{0}}\times 100$$

where F: sample fluorescence, *F*_0_: untreated liposome fluorescence, F_T_: the fluorescence of triton-treated liposomes.

### Peptide 35409 *In vitro* DNA-Binding Ability

Plasmid DNA (100 ng) alone or with peptide 35409 (0, 11, 22, 44, and 88 μM) was incubated at room temperature for 1 h (10 μL final volume). The sample was then resolved by electrophoresis on 0.5% agarose gel and stained with SYBR Green (Hsu et al., 2005; Alfred et al., 2013).

### Inhibiting DNA Synthesis *In vivo* in *E. coli* by Peptide 35409

Bacteria (15 μL, 5 × 10^6^ UFC/mL) were incubated with peptide 35409 (1× MIC and 2× MIC) at 37°C for 3 h. Then 50 μL of each sample was fixed on a slide and stained with violet crystal (1 min) and washed with water. The samples were observed by light microscopy at 100× magnification (Alfred et al., 2013). Bacteria alone or treated with ciprofloxacin [which inhibits *E. coli* DNA synthesis (Gottfredsson et al., 1995)] were used as negative and positive control of filamentation, respectively.

### Resazurine-Based Cytotoxicity Assay and Hemolytic Activity

The resazurine fluorometric test (O’Brien et al., 2000) was used for determining peptide toxicity on HeLa ATCC CCL-2 (human epidermis-derived cells) and HepG2 *ATCC* HB-8065 (human hepatocyte-derived cells) cell-lines. Cells (2 × 10^4^ per well) were transferred to 96-well plates and cultured in RPMI medium for 24 h until a monolayer was obtained. The cells were incubated with peptide 35409 (1x MIC and 2x MIC), for 72 h at 37°C. The supernatant was then skimmed off, resazurine (44 μM) added and the mixture incubated for 4 h at 37°C. Using resazurine enables cellular metabolic function to be measured, based on their oxidation state. Oxidized state is blue (cells lacking metabolic activity) and fluorescent pink in reduced state (action of oxydoreductase mainly located in viable cell mitochondria) (Perrot et al., 2003; Rolon et al., 2006).

Fluorescence was measured at 530 nm on a TECAN GENios fluorometer. RPMI medium and heat-killed bacteria (70°C) were used as negative control and untreated bacteria as positive control. IBM SPSS Statistics v20 software was used with a Tukey test for evaluating differences between treatments (*p* < 0.05 being considered statistically significant).

Hemolytic activity was determined using human RBCs. Cells were centrifuged for 15 min to remove the buffy coat and washed with PBS. Six microliter human RBC (3%) were plated into sterilized 96-well plates containing incubated peptide 35409 (serial dilutions) and PBS (200 μL final volume). After 1 h at 37°C, plates were spun at 1,000 *g* for 5 min and hemoglobin release was monitored using an ELISA plate reader (Molecular Devices), measuring absorbance at 540 nm (Almaaytah et al., 2012).

Percentage hemolysis was calculated from:

$$\%\;hemolysis=\frac{A-A_{0}}{A_{T}-A_{0}}\times 100$$

where A: sample absorbance at 540 nm, A_0_: untreated RBCs absorbance, A_T_: triton-treated RBCs absorbance.

## Results

### Helical Peptide 35409 Inhibited *E. coli* and *P. aeruginosa* Growth

**FIGURE 1:**
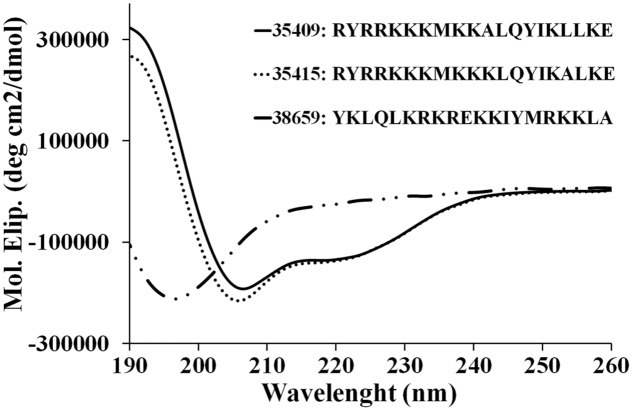
**CD spectra of peptides.** Spectra for peptides 35415, 35409, and 38659 (scrambled) were obtained by averaging three scans taken in aqueous TFE (30% v/v) solution. The results are expressed as mean residue ellipticity [Θ] in degrees per square centimeter per decimole according to [Θ] = Θλ/(100 × *l* × *c* × *n*) where Θλ represents measured ellipticity, *l* is optical path length, *c* peptide concentration, and *n* the number of aa residues in the sequence.

Peptide 35409 primary sequence contains six hydrophobic residues (bold) and 10 positively charged ones (underlined) (RYRRKKK**M**KK**AL**QY**I**K**LL**KE), meaning that is a cationic peptide (Wang and Wang, 2004). CD analysis showed that this peptide had a 190 nm maximum and two minimums at 209 and 220 nm (**Figure 1**). This data coincided with deconvolution analysis, revealing ∼90% α-helical features. Peptide 35415 was also α-helical, but scrambled peptide 38659 only had a minimum at 197 nm, suggesting the presence of random elements (**Figure 1**).

The broth dilution method revealed that peptide 35409 had antimicrobial activity against Gram-negative bacteria (MIC 22 μM against *E. coli* and MIC 44 μM against *P. aeruginosa*) and activity against Gram-positive bacteria (*S. aureus*) at greater concentration (350 μM), whilst peptides 38659 (scrambled sequence) and 35415 had no effect on bacterial growth at any concentration assayed here (**Table 1**).

**Table 1:** Peptide 35409 antibacterial activity against Gram-negative and Gram-positive bacteria.

		MIC (μM)		
		
Bacteria		35409	38659	C (-)
Gram-negative	*Escherichia coli* ML 35 (43827)	22 ± 1	G	G
	*Pseudomonas aeruginosa* 15442	44 ± 1	G	G
Gram-positive	*Staphylococcus aureus* 29213	350 ± 1	G	G


*Mean ± SD of three experiments*

*G: growth*

### Peptide 35409 Kinetic Activity

**FIGURE 2:**
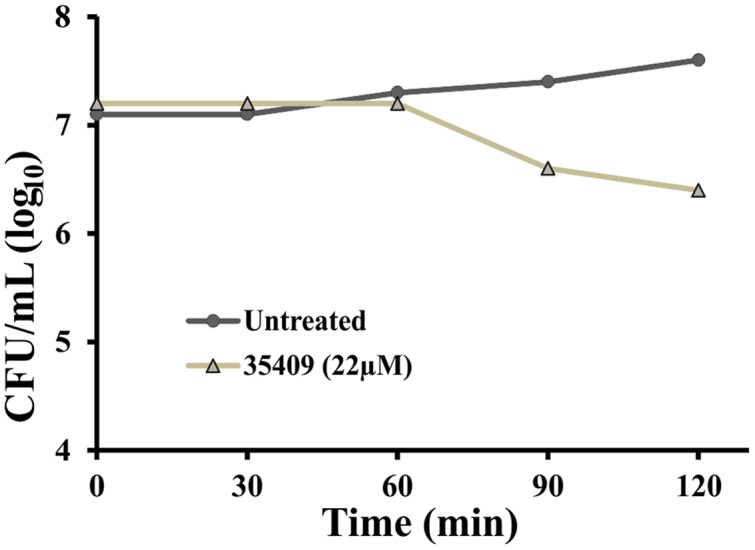
**Kinetics of peptide 35409 activity against *Escherichia coli* ML 35.** Time-dependent cell growth in the absence of peptide (●) and in the presence of 22 μM peptide 35409 (Δ). No bacterial growth was seen with peptide treatment at 22 μM and 1 log reduction was produced after 120 min, whilst growth was seen in bacteria without treatment. Data was recorded in duplicate and error did not exceed 10%.

Peptide 35409 inhibitory activity against *E. coli* ML35 cells was evaluated as regards time taken. The results showed that the peptide maintained its inhibitory activity during the time being evaluated (3 h) and reduced UFC/mL. **Figure 2** shows that the amount of UFC/mL was constant in the presence of peptide 35409 and during the first 60 min; after this, a progressive reduction in bacterial population was observed. Reading at 120 min showed that the bacterial population became reduced by 1 log compared to the initial population.

### Permeabilization of *E. coli* ML 35 Membrane

**FIGURE 3:**
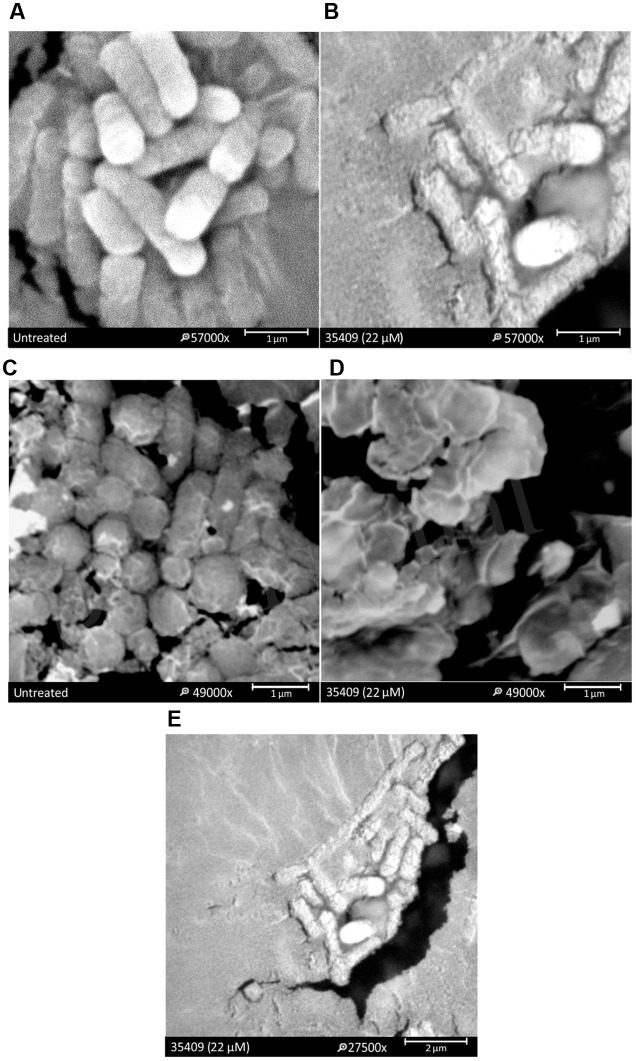
**The effect of peptide 35409 on *E. coli* ML 35 membrane.** SEM micrographies show that bacteria treated with peptide had perturbations on their membrane: **(A)** bacteria in the absence of peptide and **(B)** treated with peptide 35409. A lytic effect on exposing spheroplasts to peptide 35409 is shown: **(C)** spheroplasts in the absence of peptide and **(D)** treated with peptide 35409. Further morphological change involving bacterial elongation was observed in bacteria treated with peptide 35409 **(E)**.

The effect of peptide 35409 on *E. coli* cell envelop was evaluated by SEM. Morphological changes were observed on the surface of bacteria treated with peptide 35409, thereby indicating the deterioration of cell membrane (**Figures 3A,B**). Peptide 35409 also caused lysis in spheroplasts which are bacteria lacking external membrane and bacterial wall (**Figures 3C,D**). Interestingly, it was found that peptide 35409 caused a morphological change consisting of the lengthening of bacterial bodies (**Figure 3E**).

**FIGURE 4:**
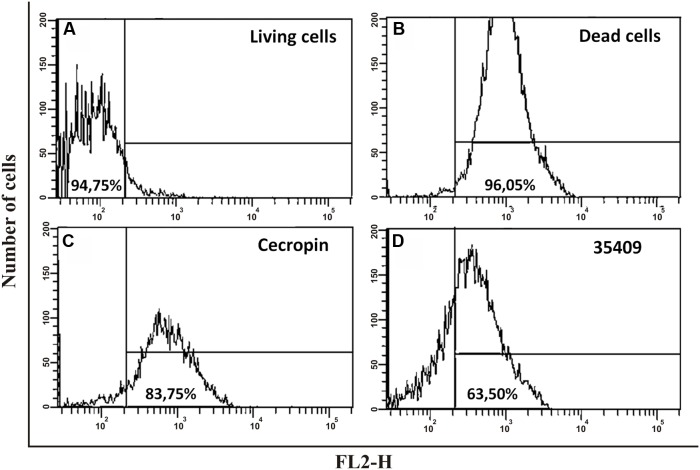
**The effect of peptide 35409 on *E. coli* ATCC 25922 integrity and viability.** 5 × 10^6^ CFU/mL were incubated for 4 h with different treatments; PI incorporation was evaluated by flow cytometry. **(A)** Cells without peptide using PI as negative control; **(B)** Heat-killed cells (5 min at 100°C and then 3 h at 70°C) with PI as positive control; **(C)** Cecropin-treated cells (12 μM); **(D)** Peptide 35409-treated cells (88 μM). Data is given in percentages (%). Events (10,000) were counted for each experiment.

Membrane permeability determination involved cells treated with peptide 35409 being stained with PI, which only enters cells having damaged cytoplasmic membranes or dead bacteria (Chau et al., 2011). PI incorporation by *E. coli* ML35 cells treated with peptide 35409 was evaluated by flow cytometry. **Figure 4** shows that dead bacteria and those treated with peptide (4x MIC) incorporated PI (63.5%), whilst bacteria without treatment did not incorporate PI. The AMP cecropin, which has been reported to induce inner membrane perturbation (Chen et al., 2003; Arcidiacono et al., 2009), induced high PI incorporation (83.75%) (**Figure 4**).

**FIGURE 5:**
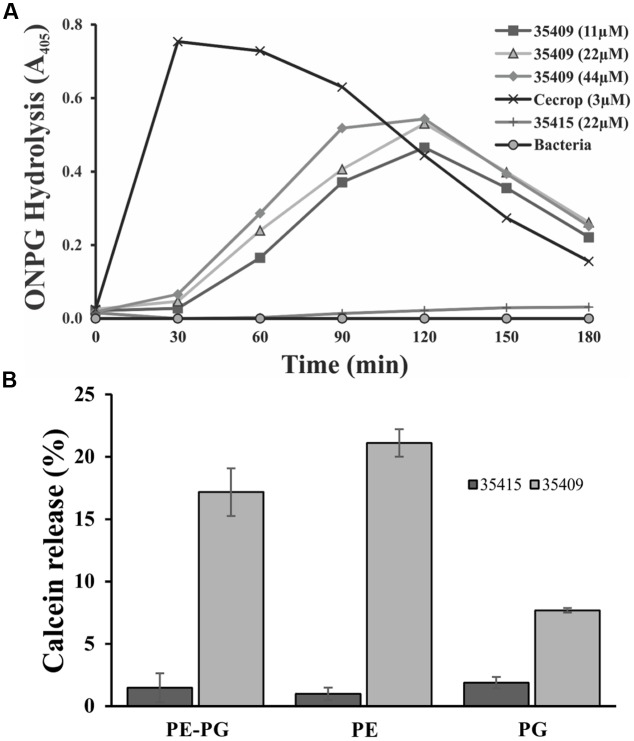
***Escherichia coli* ML 35 internal membrane permeabilization and interaction with membrane phospholipids.** **(A)** Peptide capability for permeabilizing *E. coli* ML-35 internal membrane was evaluated by using ONPG substrate and treating bacteria with peptide at different concentrations, 11, 22, 44 μM and with cecropin (3 μM) and peptide 35415 (22 μM) as positive and negative control, respectively. **(B)** Calcein-loaded liposomes were treated with peptide 35409 (22 μM) for evaluating their interaction with phospholipids from *E. coli* internal membrane. Peptide 35415 was used as negative control. Greater calcein release was seen in liposomes only composed of PE.

In another assay, ONPG hydrolysis by *E. coli* ML-35 strain cells, having no lactose permease but constitutively forming β-galactosidase (a cytoplasmic enzyme), was used for evaluating membrane permeability regarding time taken. When β-galactosidase is released it causes ONPG hydrolysis, producing yellow *o*-nitrophenol (ONP). **Figure 5A** shows that peptide 35409 (11-44 μM) caused ONP formation after 30 min (maximum at 120 min), while cecropin (3 μM) allowed more rapid ONP formation (maximum at 30 min).

### Calcein Leakage in LUVs Having Different Lipid Composition

Antimicrobial peptides activity is due mainly to membrane-permeabilization and is related to membrane lipid composition (Yeaman and Yount, 2003; Dennison et al., 2008; Teixeira et al., 2012). ONPG hydrolysis and PI incorporation assays showed that peptide 35409 permeabilized the membrane of *E. coli* ML-35 cells. To know whether peptide 35049 activity is dependent on membrane lipid composition, LUVs consisting of PE, PG, or a mixture of both PE/PG (8:2) containing calcein were prepared and treated with peptides 35409 and 35415. **Figure 5B** shows that peptide 35409 induced calcein release in liposomes having different lipid composition. Greater release (21%) was seen in liposomes consisting just of PE and lower release (8%) in liposomes just consisting of PG, whilst liposomes consisting of PE and PG (8:2) had 17 % calcein release. On the other hand, peptide 35415 (lacking inhibitory activity) had ≤2% release for all types of liposomes (**Figure 5B**).

### Peptide 35409 Binding to Bacterial DNA and Inhibiting Cell Division

**FIGURE 6:**
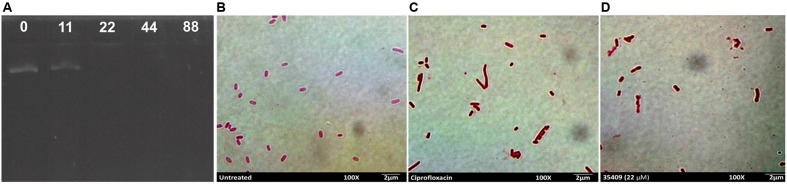
**Peptide 35409 action on bacterial DNA.** Peptide 35409-DNA binding capability was measured by gel retardation assay of plasmid DNA electrophoretic run on agarose gel. **(A)** Peptide concentrations were 0, 11, 22, 44, and 88 μM (as shown in the upper part of each well). For *in vivo* bacterial filamentation assay, **(B)** untreated bacteria were used as negative control, **(C)** ciprofloxacin-treated bacteria as positive control and **(D)** bacteria incubated with peptide 35409 (22 μM). Bacterial elongation can be seen regarding treatment with peptide 35409.

The gel retardation assay assesses peptide-DNA binding by retarding the migration of DNA bands across agarose gels (Alfred et al., 2013). It was observed that plasmid DNA was still able to migrate into the gel at peptide concentrations lower than MIC, the same as control (untreated DNA), whereas almost all the DNA remained at the origin at ≥MIC concentrations (**Figure 6A**).

It has been reported that some AMPs inhibit DNA synthesis and bacteria then grow without causing cell lysis (Falla et al., 1996). As peptide 35409 had *in vitro* DNA-binding ability then a filamentation assay was used for evaluating whether peptide 35409 could inhibit DNA synthesis *in vivo*. **Figure 6D** shows a lengthening of bacterial bodies caused by peptide 35409 treatment at MIC.

### Peptide 35409 Was Not Cytotoxic on Eukaryotic Cells

**FIGURE 7:**
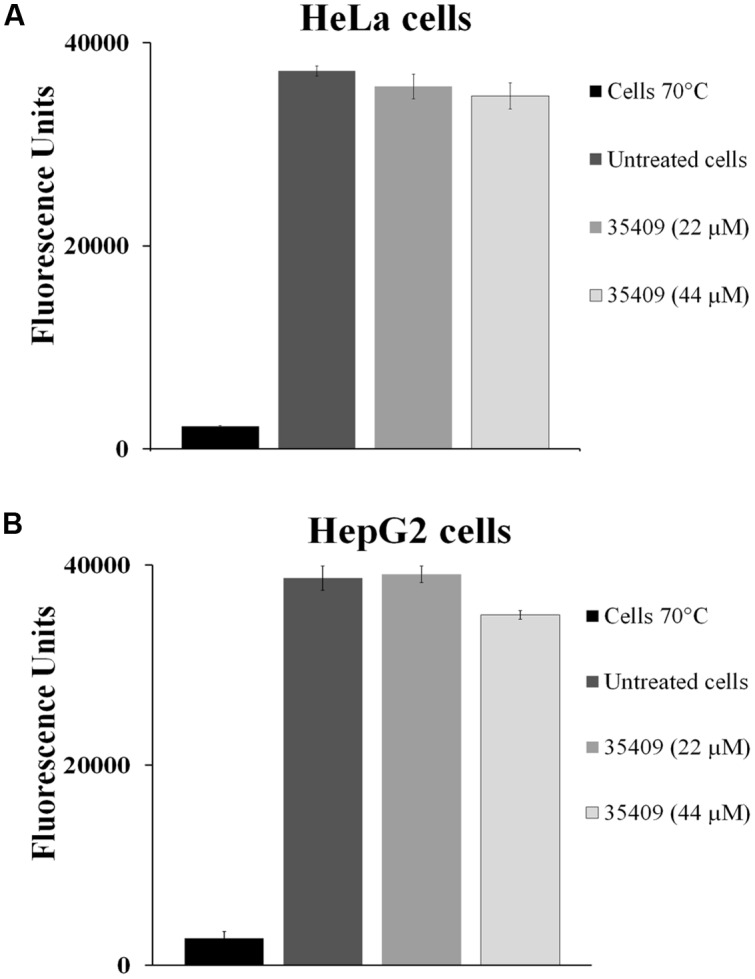
**Cytotoxicity assays.** The effect of 35409 peptide on **(A)** HeLa cells and **(B)** HepG2 cells. Both cell-lines were pre-incubated with 22 and 44 μM of peptide 35409. Cells in the absence of peptide were used as negative control and cells killed at 70°C as positive control. Data was recorded in triplicate and had less than 10% standard deviation.

A fluorometric assay using resazurine as viability indicator evaluated the toxic effect of peptide 35409 on HeLa and HepG2 cell-lines (**Figure 7**). The results revealed no statistically significant difference when treating HeLa and/or HepG2 cells with peptide 35409 (22 and 44 μM) and cells without any type of treatment. Evaluating the effect of peptide 35409 (increasing concentrations) on human RBCs revealed that the peptide lysed around 14% of the cells at the highest concentration assayed (350 μM) (data not shown).

## Discussion

The search for new agents to combat bacterial infections represents a significant focus for current research given the increased appearance of strains which are resistant to available antimicrobial drugs. AMPs have emerged as a useful alternative for combating the problem and studying this type of molecules is increasing.

Peptide 35409 sequence (RYRRKKKMKKALQYIKLLKE) has characteristics typical of some AMPs: being cationic, having α-helix structure elements (**Figure 1**) and having arginine in the sequence’s first position (Lata et al., 2010). This sequence has not been reported as being an AMP; however, it has ∼40% similarity with AMP latarcin-1 sequence. Peptide 35409 antimicrobial activity against Gram-positive and Gram-negative bacteria was analyzed here to address its possible use as target in developing a new AMP.

Peptide 35409 acted on Gram-positive and Gram-negative bacteria, inhibiting Gram-negative growth 16-fold regarding Gram-positive growth (**Table 1**). Interestingly, even though peptide 35409 and cecropin P1 isolated from pig intestine have different sequences and little similarity, the same activity pattern having preference concerning Gram-negative bacteria has been observed for both (Arcidiacono et al., 2009). It has been suggested that such pattern could have been due to differences in bacterial membranes; the peptidoglycan layer in *S. aureus* cell wall could confer greater resistance against peptide activity, as has been reported for these bacteria concerning antibiotic activity (Lemmen et al., 2004). Peptide 35409’s high α-helix structure content and peptide 38659’s random structure (**Figure 1**) (having no effect on bacterial growth) suggested that secondary structure could also be involved in or even determinant in 35409 peptide activity, as has been shown for some AMPs (Hwang and Vogel, 1998).

Peptide 35409 had a 22 μM MIC for the *E. coli* ML 35 strain according to broth dilution results. Such value fell within the range of MICs reported for the different AMPs being studied. For example, AamAP1 and synthetic CP-1 AMPs had activity against Gram-positive and Gram-negative bacteria with MIC 22-150 and 3-77 μM, respectively (Zhang et al., 2009; Almaaytah et al., 2012).

Peptide kinetic activity against *E. coli* ML 35 was constant regarding CFU/mL during the first 60 min and then became reduced, reaching 1 log at 120 min (**Figure 2**). This suggested slow kinetic action compared to that observed for other AMPs (Arcidiacono et al., 2009; Joshi et al., 2010). Even though bacterial death was observed, the results were not conclusive enough for determining whether the action was bactericidal or bacteriostatic.

The peptide’s effect on bacterial surface was evaluated by SEM to address such issue. The micrographies revealed deterioration on bacterial surface caused by this peptide (**Figures 3A,B**). The peptide also provoked lysis in bacteria devoid of external membrane and cell wall (spheroplasts); suggesting a direct effect on internal membrane. In fact, when PI incorporation and ONPG hydrolysis kinetics concerning *E. coli* ML35 cells was evaluated, it was found that the peptide slowly permeabilized inner membrane (compared to cecropin) (**Figures 4** and **5A**). There could thus be a relationship between ONPG hydrolysis kinetics and peptide 35409 kinetic activity (**Figures 2** and **5A**) since the reduction of UFC/mL occurred after 90 to 120 min had elapsed, coinciding with membrane permeabilization.

It has been described that factors such as hydrophobicity, charge, hydrophobic moment, and polar angle are related to antimicrobial and hemolytic activity, but this is not always a linear or direct association (Teixeira et al., 2012). Peptide 35409 has a +9 charge and -1.54 hydrophobicity and, in spite of being cationic, has had greater interaction with liposomes consisting of zwitterion phospholipid at physiological pH (PE) but not with liposomes consisting of negatively charged phospholipid (PG) (**Figure 5B**). This would coincide with a direct correlation between PE content on inner lipid membrane and antimicrobial activity which has been reported for some α/β helical peptides, also indicating that peptide 35409-membrane interaction did not depend on charge or electrostatic interactions (contrary to that reported for most AMPs) (Chen et al., 2003; Teixeira et al., 2012) but was rather associated with hydrophobicity and amphipathicity (Epand et al., 2005, 2007). While this experiment confirmed interaction with the most abundant phospholipid on bacterial membrane, the slow release of calcein on LUVs endorsed a transitory interaction (**Figure 5B**), thereby suggesting two possible mechanisms of action for peptide 35409. The first concerns the formation of small, short-lived pores allowing peptide translocation, and release of calcein, as described for some AMPs having similar characteristics to those of peptide 35409: short, cationic, α helical, and amphipathic (Giangaspero et al., 2001; Yan et al., 2013). The second is the translocation of the peptide favored by the abundance of PE on the inner membrane of *E. coli* (Epand et al., 2006), causing low and slow calcein release (**Figure 5B**).

Based on the forgoing and the morphological change observed by SEM (**Figure 3E**) it might be suggested that cell division became inhibited; this led to evaluating whether DNA is a target for peptide 35409 action. **Figure 6A** shows that the peptide retarded plasmid DNA electrophoretic run, suggesting an interaction between the peptide and the DNA chain. The DNA-peptide complex not only had greater weight but also lost affinity for the positive pole (anode) due to the positive charges provided by the cationic peptide. Peptide 35409 interaction with DNA could be attributed to electrostatic attraction between the peptide and the phosphate groups of DNA molecules, where the peptide’s α-helix conformation (revealed by CD for peptide 35409) plays an important role at spatial level for insertion into the DNA chain (Rivas-Santiago et al., 2006).

On the other hand, when a bacteria’s DNA synthesis is inhibited, this changes its morphology, it becomes longer without achieving cell division (morphological change called filamentation) (Subbalakshmi and Sitaram, 1998; Rosenberger et al., 2004). Peptide 35409 treated bacteria underwent such morphological change in an assay *in vivo* as observed by SEM (**Figure 3E**) and light microscopy (**Figures 6B–D**). The above, together with the results of calcein release, indicated that the peptide interacted transitorily with the bacterial membrane, affected cell entry and bound to DNA, inhibiting its synthesis and impeding cell division.

As main problem with AMPs lies in their high toxicity concerning eukaryotic cells; evaluating peptide cytotoxicity regarding eukaryote membranes is an important step in using them as bacterial agents (Koczulla and Bals, 2003; Chen et al., 2005). It was found that peptide 35409 caused hRBC lysis at MIC and that such lytic activity was concentration-dependent. However, lysis percentages were very low, thereby agreeing with that reported for various AMPs; SA-2-SA-5 has 10% maximum hemolysis at 500 μg/mL and magainin 1 has maximum 3% hemolysis at 50 μM. Other natural AMPs, such as melittin, have 100% hemolytic activity at 12 μg/mL (Maher and McClean, 2006; Joshi et al., 2010; Lewies et al., 2015). Such low hemolytic capability could be associated with a lower percentage of PE in RBC external monolayer (Daleke, 2008) since it was observed that peptide 35409 preferentially interacted with this phospholipid (**Figure 5B**).

It was also found that this peptide did not affect eukaryote cell viability (**Figures 7A,B**), thereby making it a target for further studies when looking for new therapeutic agents against infectious diseases.

The present study has thus reported a new sequence (peptide 35409) having usual AMP characteristics and double-action mechanism on *E. coli.* Its target of action is not only the bacterial membrane but also cytoplasmatic DNA. Our results suggested that helical conformation, hydrophobicity, and amphipathicity play an important role in its mechanism of action. Even though this peptide had hemolytic activity, it was low and had no toxicity regarding other eukaryotic cells which is why we consider that it is a good candidate when designing and developing new AMPs having selectivity for bacterial membranes.

## Author Contributions

HC, GA-P, DS, and AB-S conceived and designed the experiments; AB-S, DS, CH, GA-P performed the experiments and analyzed the data; WP, MP contributed reagents/materials/analysis tools; AB-S, GA-P, HC wrote the paper.

## Conflict of Interest Statement

The authors declare that the research was conducted in the absence of any commercial or financial relationships that could be construed as a potential conflict of interest.
