# Supplementary material for: A New Synthetic Peptide Having Two Target of Antibacterial Action in E. coli ML35
Source: Front Microbiol. 2016 Dec 20;7:2006. doi: 10.3389/fmicb.2016.02006 (PMC5167725; doi:10.3389/fmicb.2016.02006)
Supplement: Supplementary file 1 [file Key_Concept_List.PDF]

Key concept list→

1. **Liposome:** this is a vesicular and colloidal structure, consisting of one or more phospholipid bilayers around an equal amount of aqueous compartments (Shashi, 2012). The size of these vesicles varies from 25 nm to 10 µm in diameter (Berger et al., 2001).
   Liposomes can be used for studying different substances interactions with cell membranes, for example, between antimicrobial peptides and bacterial cells (Soblosky et al., 2015). Their usefulness in such type of study lies the easy of obtaining vesicles having different phospholipid composition and the simplicity of assays leading to understanding of a membrane's individual lipid components affecting such interaction (Epand et al., 2006).

   Phospholipid: a phospholipid is an amphipathic molecule formed by a phosphate group (polar heads), a glycerol and a fatty acid chain (hydrophobic tails) (Devlin, 2002).

2. **LUV:** a LUV or large unilamellar vesicle is a large, easily obtained liposome, having a single lipid bilayer. Its size ranges from 100 to 1,000 nm in diameter (Akbarzadeh et al., 2013).

3. **Secondary structure:** is the structure a peptide or protein assumes as a result of becoming twisted. Secondary structure arises from the interaction between neighbouring amino acids in a sequence by hydrogen bonds (Aydin and Technology, 2008).

4. **Hydrophobicity:** a chemical compound's hydrophobicity is related to its transfer free energy from a polar medium to a non-polar medium. A peptide's total hydrophobicity is calculated by adding the transfer free energy of each amino acid in its sequence. This property can provide information regarding how peptides interact with membranes during key biological events (Pirtskhalava et al., 2013).

5. **Amphipathicity:** when a molecule has two separate parts, one lipophilic and the other hydrophilic, this is considered amphipathic or amphiphilic. This characteristic gives a molecule with a preference for remaining in the interface between polar and nonpolar environments (Pirtskhalava et al., 2013). Peptides can have an amphiphilic structure due to a certain distribution of polar and non-polar amino acids throughout a chain, or can adopt a secondary structure favouring such property, this being an important factor for interacting with membranes (Kaiser and Kezdy, 1983).

6. **Net charge:** a molecule's net charge is the sum total of its positive and negative charges, i.e. the relationship between protons and neutrons within a molecule (Schafer and Association, 1992; Zumdahl and DeCoste, 2010). Most antimicrobial peptides are cationic (positively charged) at physiological pH (Yeaman and Yount, 2003; Téllez, 2010).

7. **Permeabilisation:** is damage or perforation allowing the free exchange of internal and external components.
   It is defined as a process alleviating cell membrane or wall's permeability barrier permitting the free mobility of substances across the cell wall or membrane. This includes substrates and products (Sridhar, 2008).

8. **Cytotoxicity:** refers to a chemical compound's (food, cosmetic, o drug) or mediating cell (for example, cytotoxic T-cells) capability to cause cell death regardless of the mechanism of death so produced (Roche, 2008).

References:

Akbarzadeh, A., Rezaei-Sadabady, R., Davaran, S., Joo, S.W., Zarghami, N., Hanifehpour, Y., et al. (2013). Liposome: classification, preparation, and applications. *Nanoscale Res Lett* 8(1)**,** 102. doi: 10.1186/1556-276X-8-102.

Aydin, Z., and Technology, G.I.o. (2008). *Bayesian Models and Algorithms for Protein Secondary Structure and Beta-sheet Prediction.* Georgia Institute of Technology.

Berger, N., Sachse, A., Bender, J., Schubert, R., and Brandl, M. (2001). Filter extrusion of liposomes using different devices: comparison of liposome size, encapsulation efficiency, and process characteristics. *International journal of pharmaceutics* 223(1)**,** 55-68.

Devlin, T.M. (2002). *Textbook of Biochemistry with Clinical Correlations.* Wiley.

Epand, R.F., Schmitt, M.A., Gellman, S.H., and Epand, R.M. (2006). Role of membrane lipids in the mechanism of bacterial species selective toxicity by two alpha/beta-antimicrobial peptides. *Biochim Biophys Acta* 1758(9)**,** 1343-1350. doi: 10.1016/j.bbamem.2006.01.018.

Kaiser, E.T., and Kezdy, F.J. (1983). Secondary structures of proteins and peptides in amphiphilic environments. (A review). *Proc Natl Acad Sci U S A* 80(4)**,** 1137-1143.

Pirtskhalava, M., Vishnepolsky, B., and Grigolava, M. (2013). Transmembrane and antimicrobial peptides. Hydrophobicity, amphiphilicity and propensity to aggregation. *arXiv preprint arXiv:1307.6160*.

Roche (2008). "Apoptosis, Cytotoxicity and Cell Proliferation", (ed.) R. H.).

Schafer, L.E., and Association, N.S.T. (1992). *Taking Charge: An Introduction to Electricity.* National Science Teachers Association.

Shashi, K. (2012). A complete review on: liposomes. *IRPJ* 3(7)**,** 1-16.

Soblosky, L., Ramamoorthy, A., and Chen, Z. (2015). Membrane interaction of antimicrobial peptides using E. coli lipid extract as model bacterial cell membranes and SFG spectroscopy. *Chem Phys Lipids* 187**,** 20-33. doi: 10.1016/j.chemphyslip.2015.02.003.

Sridhar, K.R. (2008). *Frontiers in Fungal Ecology, Diversity and Metabolites.* I.K. International Publishing House Pvt. Limited.

Téllez, G., Castaño JC. (2010). Péptidos antimicrobianos. *Infectio* 14(1)**,** 55-67.

Yeaman, M.R., and Yount, N.Y. (2003). Mechanisms of antimicrobial peptide action and resistance. *Pharmacological reviews* 55(1)**,** 27-55.

Zumdahl, S.S., and DeCoste, D.J. (2010). *Introductory Chemistry.* Cengage Learning.
